# Sensorimotor and Pain Modulation Brain Abnormalities in Trigeminal Neuralgia: A Paroxysmal, Sensory-Triggered Neuropathic Pain

**DOI:** 10.1371/journal.pone.0066340

**Published:** 2013-06-18

**Authors:** Danielle D. DeSouza, Massieh Moayedi, David Q. Chen, Karen D. Davis, Mojgan Hodaie

**Affiliations:** 1 Division of Brain, Imaging and Behaviour- Systems Neuroscience, Toronto Western Research Institute, University Health Network, Toronto, Canada; 2 Institute of Medical Science, University of Toronto, Toronto, Canada; 3 Division of Neurosurgery, Toronto Western Hospital & University of Toronto, Toronto, Canada; The Hebrew University Medical School, Israel

## Abstract

**Objective:**

Idiopathic trigeminal neuralgia (TN) is characterized by paroxysms of severe facial pain but without the major sensory loss that commonly accompanies neuropathic pain. Since neurovascular compression of the trigeminal nerve root entry zone does not fully explain the pathogenesis of TN, we determined whether there were brain gray matter abnormalities in a cohort of idiopathic TN patients. We used structural MRI to test the hypothesis that TN is associated with altered gray matter (GM) in brain areas involved in the sensory and affective aspects of pain, pain modulation, and motor function. We further determined the contribution of long-term TN on GM plasticity.

**Methods:**

Cortical thickness and subcortical GM volume were measured from high-resolution 3T T1-weighted MRI scans in 24 patients with right-sided TN and 24 healthy control participants.

**Results:**

TN patients had increased GM volume in the sensory thalamus, amygdala, periaqueductal gray, and basal ganglia (putamen, caudate, nucleus accumbens) compared to healthy controls. The patients also had greater cortical thickness in the contralateral primary somatosensory cortex and frontal pole compared to controls. In contrast, patients had thinner cortex in the pregenual anterior cingulate cortex, the insula and the orbitofrontal cortex. No relationship was observed between GM abnormalities and TN pain duration.

**Conclusions:**

TN is associated with GM abnormalities in areas involved in pain perception, pain modulation and motor function. These findings may reflect increased nociceptive input to the brain, an impaired descending modulation system that does not adequately inhibit pain, and increased motor output to control facial movements to limit pain attacks.

## Introduction

Idiopathic trigeminal neuralgia (TN) is a chronic neuropathic pain disorder characterized by recurring highly intense electric shock-like pain in the distribution of one or more branches of the trigeminal nerve. The pain is usually unilateral, occurring either spontaneously or precipitated by innocuous sensory stimuli and movements. Although some sensory abnormalities have been reported in TN using detailed quantitative sensory testing [1], [2], there is typically no major sensory loss detectable with bedside clinical tests, as observed in other neuropathic pains [1], [3]. For example, in a large multi-center study examining somatosensory abnormalities in different neuropathic pain syndromes, typical sensory abnormalities in TN were mechanical hyperalgesia without sensory loss; an uncommon combination in all other syndromes examined [1].

Many theories have been proposed to explain the unique pain characteristics of idiopathic TN. In general, theories of TN focus on either central or peripheral nervous system pathogenesis [4]. Given that patients with TN are typically initially responsive to anticonvulsant medications, it has been suggested that the pain paroxysms characteristic of TN originate in the central nervous system and are analogous to epileptic seizures in brainstem trigeminal structures [4], [5]. Alternatively, proponents of a peripheral etiology suggest that TN results from damage to the trigeminal nerve or its myelin, with the most prevalent theory proposing neurovascular compression of the trigeminal nerve root entry zone (REZ) by a nearby arterial branch [3]. Although neurovascular contact (NVC) or compression of the trigeminal nerve is frequently observed, a significant number of idiopathic TN cases do not demonstrate clear evidence of neurovascular compression [6]. As the putative pathogenesis of TN is unique, so are its clinical features. While tumors or other structural abnormalities can result in the perception of a trigeminal neuropathic pain, characterized by persistent pain and sensory changes, idiopathic TN patients are mostly pain-free between attacks and without major sensory abnormalities [7]. Since NVC or compression alone does not fully explain this unique syndrome, central abnormalities may significantly contribute to TN pain [8], [9].

Patients with chronic pains other than TN can show abnormal gray matter (GM) in brain regions associated with pain and its modulation, sensory-discriminative and cognitive-affective dimensions of pain, emotion, and motor function [10], [11], [12], [13], the latter of which may be related to alterations in muscular activity aimed at limiting movements to protect the system from further injury and support healing [14], [15]. Therefore, GM abnormalities may reflect maladaptive plasticity arising from long-term pain and/or nociceptive input to the brain [16], impaired pain modulation [17], and/or compensatory/nocifensive motor behaviors to avoid triggering pain [15]. Importantly, while most chronic pain syndromes are also accompanied by prominent sensory deficits, TN is predominantly characterized by pain, but not major sensory loss [1]. This provides a unique window to our understanding of how pain impacts central GM. GM abnormalities may be dynamic, progressive, and pain-driven over time [10], [12], [18]. Based on the described studies and in consideration of the unique features of neuropathic pain in TN, we hypothesized that compared to healthy controls TN patients would have GM (i) increases in areas associated with pain perception and emotion (*e.g*., thalamus, primary and secondary somatosensory cortex (S1, S2), amygdala, anterior and mid-cingulate cortex (ACC; MCC), insula and prefrontal cortex (PFC), (ii) decreases in areas associated with pain modulation (*e.g*., periaqueductal gray (PAG)), (iii) increases in motor regions (*e.g*., primary motor cortex (M1), basal ganglia), and (iv) abnormalities that progress due to pain chronicity.

## Methods

### Ethics Statement

The University Health Network Research Ethics Board approved this retrospective study of idiopathic TN patients. There was no active participation in this study by patients, as patient data were analyzed retrospectively. Additionally our Research Ethics Board provides study approval, but does not require individual patient consent for retrospective studies. All scans were anonymized prior to analysis and stored in secure databases. Recruitment of healthy control subjects and the procedure whereby their consent was obtained was also approved by the University Health Network Research Ethics Board. Each healthy control participant provided written informed consent.

### Participants

Twenty-four right-handed idiopathic TN patients from the Toronto Western Hospital seen between May 2008 and February 2011 were included in the study. Inclusion criteria were: (1) unilateral (right-sided) pain involving at least the maxillary or mandibular (V2 or V3) branches of the trigeminal nerve, (2) intense, sharp, superficial or stabbing paroxysmal facial pain precipitated from trigger areas or by trigger factors; stereotyped attacks for each patient; no clinically evident neurological or sensory deficit or not attributed to another disorder [19], and (3) no previous surgical procedures for TN. Demographic and clinical details for patients were obtained via retrospective chart reviews. Patients were sex-matched to a cohort of 24 healthy pain-free control participants and the mean ages between the groups were not statistically different (*p* = 0.81, see results). At the individual level, all but 2 control participants were within 3 years of the patients.

### Imaging Protocol

Brain imaging data were acquired using a 3T GE Signa HDx MRI system fitted with an eight-channel phased array head coil. T1-weighted 3D FSPGR axial images were obtained from the top of the head to the upper cervical levels of C1–C2 (0.9×0.9×1.0 mm^3^ voxels derived from a 256×256 matrix and field of view of 24 cm (controls) or 22 cm (patients), echo time = 5 ms, repetition time = 12 ms, inversion time = 300 ms).

### Cortical Thickness Analysis

**Figure 1:**
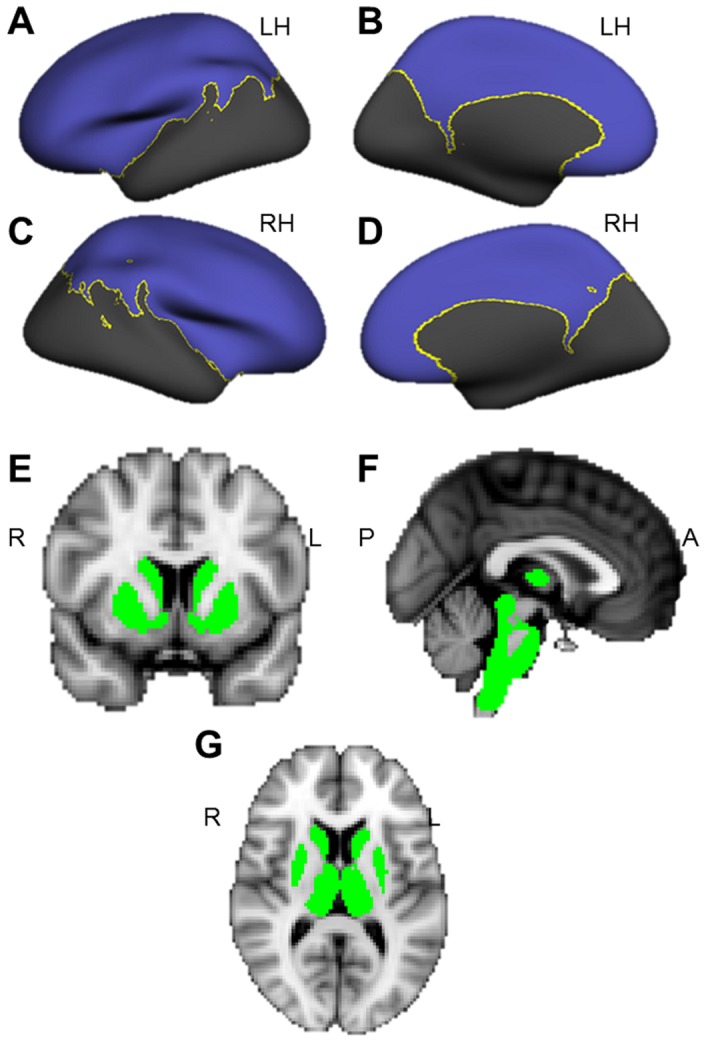
Analyses were restricted to gray matter masks. CTA and VBM gray matter analyses were restricted to masks. (**A–D**) Cortical thickness masks for the left (A & B) and right (C & D) hemispheres, including lateral (A & C) and medial (B & D) views. (**E–G**) VBM masks of subcortical structures in coronal (E), sagittal (F) and axial (G) views.

To examine cortical GM, cortical thickness analysis (CTA) was performed using FreeSurfer software v. 4.5.0 (http://surfer.nmr.mgh.harvard.edu/); methods have been described in detail elsewhere [20]. Briefly, T1-weighted scans were registered to the Talairach atlas [21]. Images underwent intensity normalization to identify tissue types, and the skulls were removed. White matter (WM) voxels were used to estimate the GM/WM boundary, which was deformed outward to the GM/cerebrospinal fluid (CSF) boundary. Thickness was computed as the distance measured between these boundaries at every point in each hemisphere. A general linear model (GLM) was used to evaluate group differences, with age included as a variable of no interest. We restricted analyses to a mask including our hypothesized regions of interest (ROI) (Fig. 1A–D) created with a predefined cortical parcellation scheme (aparc2005) implemented in FreeSurfer [22]. Data were smoothed with a 6 mm full-width half-maximum (FWHM) spatial smoothing kernel, and thresholded at a corrected *p*<0.05 (from a combination of an uncorrected image-wide *p*-value of 0.01, and a cluster threshold of 183 and 188 contiguous vertices for left and right hemispheres, respectively, based on Monte Carlo simulations with 5000 iterations (AlphaSim, implemented in Analysis of Functional NeuroImages (AFNI) (http://afni.nimh.nih.gov/afni), as done previously [10], [12], [23].

### Voxel-Based Morphometry

To examine subcortical GM group differences, we used voxel-based morphometry (VBM) [24] in FMRIB’s Software Library v. 4.1.8 (http://www.fmrib.ox.ac.uk/fsl/). Non-brain tissues were removed from structural images using the Brain Extraction Tool (BET) [25]. Tissue-type segmentation classified tissue using FMRIB’s Automated Segmentation Tool (FAST4) [26]. The GM-classified tissue was aligned to the Montreal Neurologic Institute (MNI) 152 standard 2 mm template (voxel size = 2×2×2 mm) using a linear registration tool [27]. Images were averaged creating a study-specific template, to which GM images were non-linearly re-registered. Jacobian modulation allowed group differences in GM to be reported as normalized volumes. The modulated images were then smoothed with an isotropic Gaussian kernel with a sigma of 2 mm (FWHM = 4.6 mm). Using the Harvard-Oxford subcortical structural atlas, a mask was constructed to restrict analyses to the thalamus, amygdala, hippocampus, basal ganglia, and brainstem (Fig. 1E–G). A voxelwise GLM was applied with age as a variable of no interest. Statistical significance was determined using permutation-based non-parametric testing and thresholded at *p*<0.05 using Randomise, implemented in FSL, corrected for multiple comparisons with threshold-free cluster enhancement [28]. For consistency with the CTA, VBM results were converted from MNI to Talairach coordinates using the Yale Nonlinear MNI to Talairach Conversion Algorithm [29] implemented on the BioImage Suite 2.0 website (http://www.bioimagesuite.org/Mni2Tal/).

We performed a secondary analysis to determine if prolonged nociceptive activity (i.e., pain duration) was related to GM changes. In this analysis, TN duration was treated as a regressor of interest. Analyses were restricted to the CTA and VBM masks described above. The same statistical correction methods were used to determine significant correlations between GM and pain duration.

## Results

### Patient Demographics

Patient demographic information is shown in Table 1. The TN group (n = 24) was comprised of 15 women and 9 men (mean age ± SD: 48.5±12.7 years) who had right-sided TN pain for 6.3±3.0 years with a mean age at pain onset of 43.0±12.9 years. The majority of patients were on medications for TN at the time of scan acquisition, with the most common being carbamazepine (Table 1). The healthy control group consisted of 15 women and 9 men with a mean age ± SD of 47.6±12.3 years, which was not significantly different (*p* = 0.81) from the patient group.

**Table 1:** TN Patient Demographic Information.

Patient	Sex	Age	Pain Dist	Pain Duration (years)	Age at Pain Onset	Medication
R1	F	45	V2	10	35	CBZ
R2	M	67	V2, V3	8	59	CBZ
R3	F	62	V3	11	51	CBZ, PGB
R4	F	68	V2, V3	3	65	CBZ, GBP
R5	M	52	V1, V2	5	47	CBZ
R6	F	44	V2, V3	2	42	None
R7	F	55	V2	6	49	None
R8	F	40	V2	10	30	CBZ
R9	M	50	V1, V2, V3	6.5	44	–
R10	F	63	V2	8	55	CBZ
R11	M	47	V3	5	42	CBZ, PGB
R12	F	52	V3	5	47	CBZ, VPA, TCA
R13	F	55	V2, V3	2	53	CBZ, GBP
R14	F	41	V1, V2, V3	7	34	CBZ
R15	M	24	V2	4	20	CBZ
R16	M	37	V3	1	36	CBZ, PGB
R17	M	24	V3	2	22	CBZ, TCA-N
R18	M	38	V2	2	36	CBZ
R19	F	42	V2, V3	2	40	CBZ, GBP
R20	F	52	V1, V2	3	49	CBZ
R21	F	27	V2, V3	13	14	CBZ, GBP
R22	F	56	V3	6	50	CBZ
R23	M	62	V1, V2, V3	3	59	PGB
R24	F	60	V2	6	54	CBZ

Note: – indicates information not available.

Abbreviations: Pain Dist: pain distribution, referring to the peripheral branches of the trigeminal nerve (V1: ophthalmic branch; V2: maxillary branch; V3: mandibular branch); CBZ: carbamazepine; PGB: pregabalin; GBP: gabapentin; TCA: tricyclic antidepressant; VPA: valproic acid; TCA-N: nortriptyline;

### Regions of Abnormal Cortical Thickness in TN Patients

**Figure 2:**
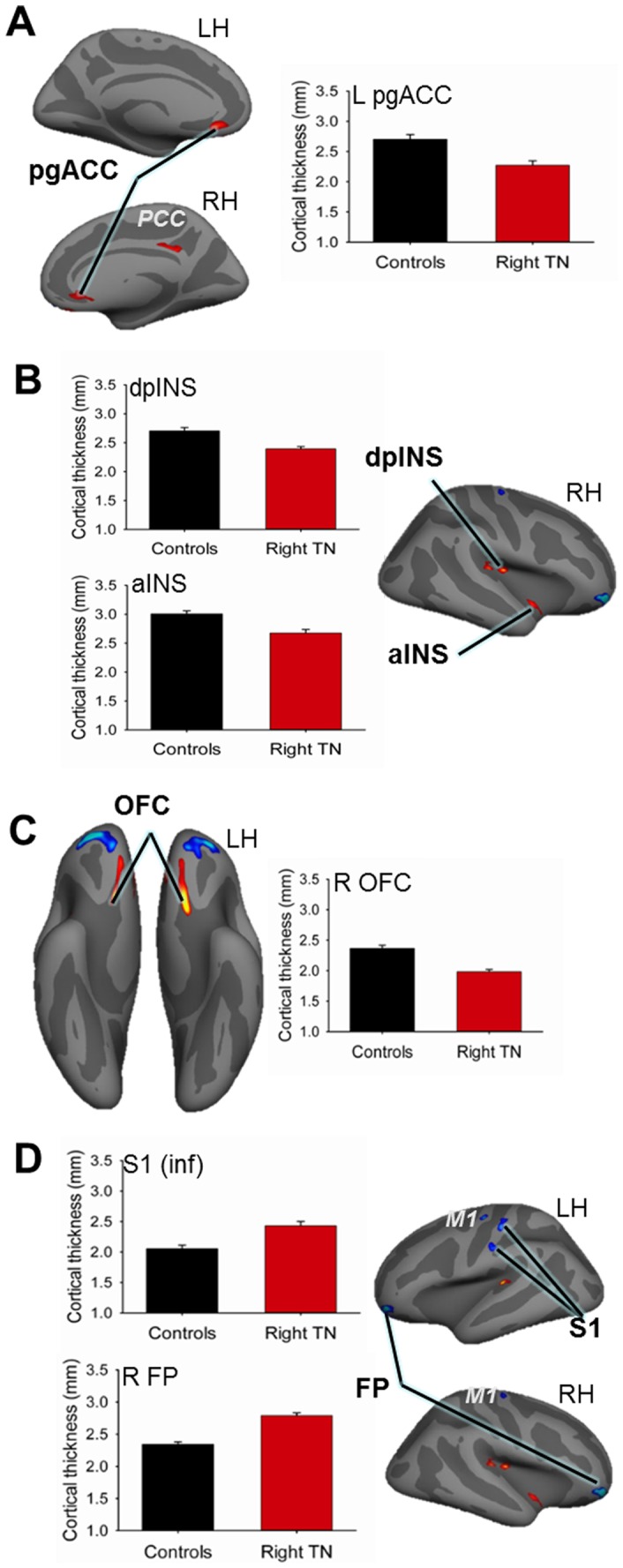
Cortical thickness abnormalities in trigeminal neuralgia patients. CTA revealed significant group differences in several cortical brain regions. Red clusters indicate thinner cortex in patients compared to controls (*p*<0.05, corrected). Prominent findings of cortical thinning in TN are shown in panels A–C, including graphs of mean cortical thickness values ± SEM (in mm): controls (black bars), patients (red bars). Areas of cortical thickening in TN are highlighted in panel D. Thinner cortex in TN was observed in: (**A**) the bilateral pgACC; graph illustrates thickness for left cluster; (**B**) the right insular cortex including the dpINS and the ventral aINS, and (**C**) the bilateral ventral OFC; graph illustrates thickness for right OFC cluster. TN patients had thicker cortex in (**D**) the bilateral FP and M1, and contralateral (left) S1; graphs illustrate thickness for right FP cluster and the inferior S1 cluster (putative face area). Abbreviations: LH =  left hemisphere; RH =  right hemisphere; R =  right; L =  left; pgACC =  pregenual anterior cingulate cortex; PCC =  posterior cingulate cortex; aINS =  anterior insula; dpINS =  dorsal posterior insula; OFC =  orbitofrontal cortex; FP =  frontal pole; M1 =  primary motor cortex; S1 =  primary somatosensory cortex.

All CTA results are provided in Table 2 and prominent findings are illustrated in Figure 2. Compared to controls, TN patients had 14–16% greater cortical thickness within three regions: the left (contralateral to side of pain) S1 including the putative face area (Fig. 2D, inferior S1 cluster), frontal pole (FP) (BA 10) bilaterally, and M1 bilaterally. CTA also revealed several cortical regions that were 10–19% thinner compared to controls. The most prominent areas of thinning were located bilaterally in the pregenual ACC (pgACC) (BA 24) and the ventral orbitofrontal cortex (OFC) (BA 11) (Fig. 2A & C). Cortical thinning was also observed in the right dorsal posterior insula (dpINS), ventral anterior insula (aINS) (Fig. 2B), and posterior cingulate cortex (PCC).

**Table 2:** Cortical Thickness Abnormalities in TN.

Hemis	Region	BA	Peak Coordinates (TAL)			# Vert	Peak T-Score	% Change	Change (*cf.* Controls)
			X	Y	Z				
Right (ipsi)	FP	10	29	53	−11	770	9.12	16.0	incr
	OFC	11	14	20	−13	418	4.86	−17.2	decr
	pgACC	24	8	29	−9	203	2.57	−13.2	decr
	PCC	23	7	−39	30	305	2.85	−9.5	decr
	S. temp	42	39	−31	12	295	3.55	−12.3	decr
	dpINS		32	−20	16	362	4.46	−12.9	decr
	aINS		41	−10	−12	206	3.15	−12.3	decr
	M1	4	25	−13	64	205	3.32	12.1	incr
Left (contra)	FP	10	−32	52	−11	680	6.59	15.8	incr
	OFC	11	−14	20	−14	490	6.38	−19.3	decr
	pgACC	24	−10	32	−10	261	3.41	−18.8	decr
	S. temp	42	−40	−32	7	232	5.50	−12.6	decr
	S1	2	−54	−17	34	232	3.68	15.5	incr
	S1	1	−47	−19	55	400	4.06	14.2	incr
	MI	4	−35	−11	55	223	4.06	13.7	incr

Abbreviations: TN: trigeminal neuralgia; Hemisp: hemisphere; BA: Brodmann Area; TAL: Talairach; # Vert: number of vertices; ipsi: ipsilateral to side of pain; contra: contralateral to side of pain; GM: gray matter; OFC: orbitofrontal cortex; PCC: posterior cingulate cortex; pgACC: pregenual anterior cingulate cortex; S. temp: superior temporal cortex; MI: primary motor cortex; S1: primary somatosensory cortex; FP: frontal pole; dpINS: dorsal posterior insula; aINS: anterior insula; % Change: percent thickness change; Change *cf.* Controls: direction of change compared to controls.

### Larger Subcortical Volumes in TN Patients

**Figure 3:**
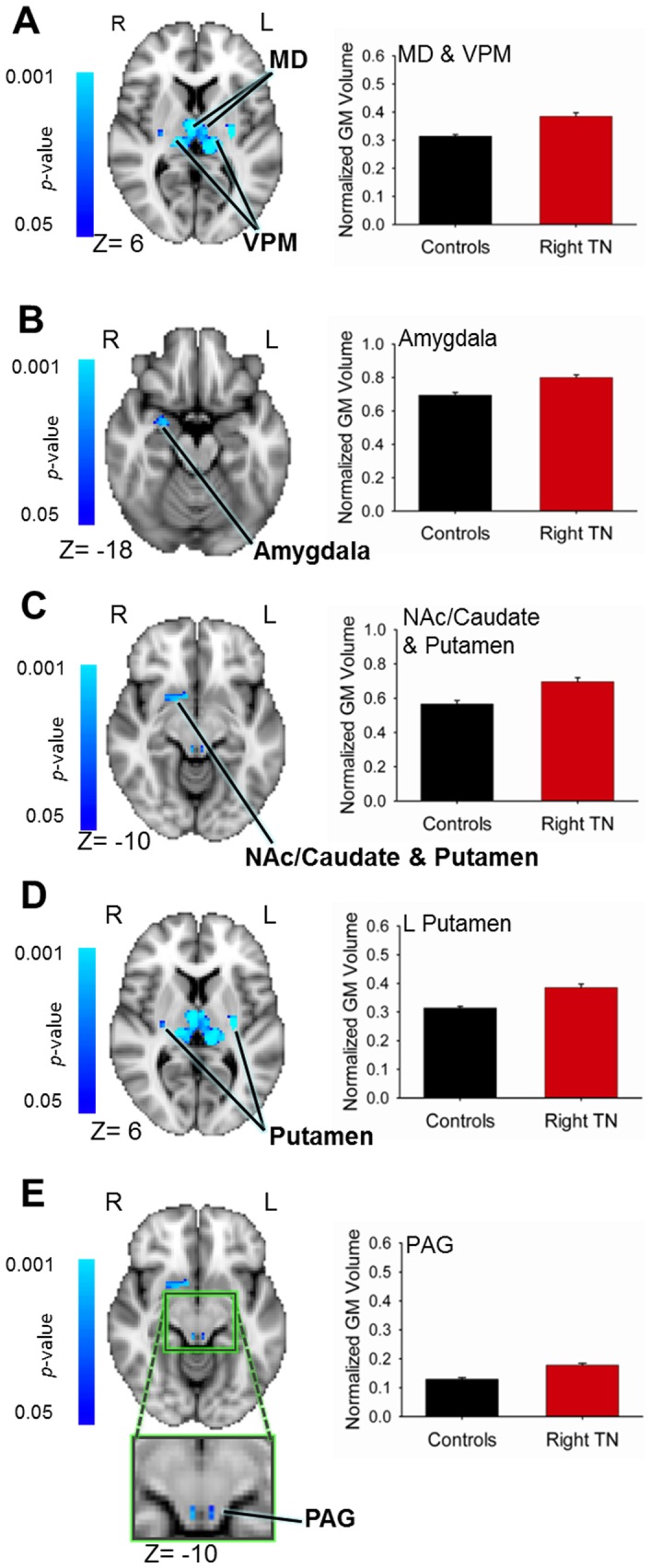
Regions of greater subcortical volume in patients with trigeminal neuralgia. VBM analysis revealed significant group differences in several subcortical brain regions. Significant results (*p*<0.05, corrected) are displayed on the MNI152 (2 mm) T1 brain template. Blue clusters indicate greater GM volume in patients compared to controls. Graphs of normalized GM volumes ± SEM are shown to the right of brain images: controls (black bars), patients (red bars). Increased patient GM volume was observed in: (**A**) the sensory thalamus, including the MD and VPM thalamus bilaterally; (**B**) the right amygdala; (**C**) a cluster spanning the right nucleus accumbens, anterior putamen and caudate; (**D**) the posterior putamen bilaterally; (**E**) the PAG (green box shows magnified region). Abbreviations: R =  right; L =  left; MD =  medial dorsal nucleus (thalamus); VPM =  ventral posterior medial nucleus (thalamus); NAc =  nucleus accumbens; PAG =  periaqueductal gray.

Subcortical regions that had significantly different GM volumes in the TN patients compared to controls are listed in Table 3. Although the ventral pons had significantly less GM volume in the TN group compared to controls, for all other subcortical regions that were significantly different between groups, patients had larger volumes. The TN group had greater GM volumes than controls bilaterally in the several thalamic regions that spanned the medial dorsal (MD) nucleus, the ventral posteromedial (VPM) nucleus (Fig. 3A), the pulvinar, and the ventral lateral (VL) nucleus. The TN group also had greater GM than controls in the right amygdala (Fig. 3B), an increase of 13%, and in a cluster spanning the nucleus accumbens (NAc), caudate, and anterior putamen (19% increase) (Fig. 3C). Bilaterally, a substantially greater volume of the posterior putamen (average increase of 38%) and in the PAG (increase of 27%) (Fig. 3D & E) was observed in the TN group compared to the control group.

**Table 3:** Subcortical Volume Abnormalities in TN.

Region	Peak Coordinates (TAL)			# Voxels	Peak T-Score	% Change	Change (*cf.* Controls)
	X	Y	Z				
Thalamus	5	−9	3	518	5.52	18.4	incr
R NAc/Caud/Putamen	7	5	−5	89	4.83	18.6	incr
L Post. Putamen	−27	−19	8	64	7.42	41.2	incr
R Post Putamen	29	−21	5	60	5.00	34.2	incr
R Amygdala	23	−5	−12	19	4.92	13.2	incr
PAG	4	−33	−6	19	5.93	27.3	incr
Pons	9	−26	−34	561	4.49	−84.9	decr

Abbreviations: TN: trigeminal neuralgia; TAL: Talairach; # Voxels: number of voxels; R: right; L: left; NAc: nucleus accumbens; Post: posterior; PAG: periaqueductal gray; % Change: percent thickness change; Change *cf.* Controls: direction of change compared to controls.

Note- voxel size for VBM analysis = 2×2×2 mm.

### No Correlation between Gray Matter Abnormalities and Pain Duration

TN pain duration was not significantly related to cortical thickness or subcortical volume for any region within the CTA and VBM masks, nor within areas of GM abnormality when specifically examined (*p*>0.05).

## Discussion

In this study, we provide novel evidence that TN, a neuropathic pain thought to arise from a peripheral event, characterized primarily by pain and the absence of major sensory loss is associated with pronounced alterations in brain GM. These abnormalities occur in neuroanatomical regions that contribute to sensory-discriminative and cognitive-affective dimensions of pain, pain modulation, and motor function. Specifically, we identified that compared to controls, TN patients have: (1) greater GM in the thalamus, contralateral S1 (putative face area), amygdala, FP, PAG, M1, and basal ganglia (including the putamen and NAc), and (2) cortical thinning in the OFC, pgACC, and insula. Abnormalities in these brain regions have been reported in other chronic pain disorders [13]. However, in previous studies, the contributions of sensory loss and pain are often intertwined. Our findings raise the possibility that the CNS contributes to the development and/or maintenance of TN pain and help advance our knowledge of how a peripheral event is associated with central gray changes.

Potential mechanisms underlying MR-detectable GM differences have been broadly divided into two categories: neuronal and non-neuronal mechanisms, including alterations in vasculature, the size or numbers of glia and/or dendritic spines/branches [30], [31]. Evidence for the activity-dependent alteration of these structures has been demonstrated as a result of experience and learning, with the majority of studies reporting non-neuronal mechanisms as the primary contributor to GM differences in regions outside of the hippocampus [31]. GM abnormalities in TN patients may also occur via these mechanisms, elicited by experience-dependent factors such as changes in nociceptive input to the CNS, affect, and learned compensatory/nocifensive motor behaviors. Alternatively, it is possible that some individuals are more susceptible to developing chronic pain because of pre-existing GM abnormalities [13]. This possibility cannot explicitly be tested in a cross-sectional study. In the current study, we did not find any evidence to support this possibility based on assessing disease duration-related GM abnormalities. However, we cannot totally rule out the contribution of some pre-existing functional or structural vulnerability as was found in other chronic pain studies [13], [32].

Consistent with our *a priori* hypothesis, we found that TN patients had more GM in the thalamic nuclei compared to controls. Although we included patients with only right-sided pain, the greater thalamic volume was found bilaterally. This bilateral finding is in line with a previous functional MRI study on TN patients that showed bilateral thalamic activity when unilateral stimuli were used to evoke pain attacks [33]. Also, certain thalamic neurons, including MD neurons, have been shown to have large, bilateral receptive fields [34], which may in part account for this finding. Previous studies of chronic facial pain have reported mixed findings with regard to patient thalamic volume relative to controls [11], [12], [18]. This variability may be due to the heterogeneity of pain symptomology between chronic facial pains [35]. For example, patients with trigeminal neuropathic pain have constant pain accompanied by numbness [36]. Indeed, one study examining different chronic facial pains found that only trigeminal neuropathy patients had significantly smaller thalamic volume compared to controls [11]. It is possible that this difference reflects a system that is steadily receiving nociceptive, but not discriminative touch input, fitting with trigeminal neuropathy symptomology. However, a barrage of nociceptive input, in combination with mostly intact discriminative touch sensations (i.e., no numbness), may induce GM increases in the medial and lateral thalamic nuclei of TN patients. Similarly, this theory may account for our finding of a thicker putative face S1 in TN. The barrage of nociceptive input to the thalamus from the trigeminal nerve may in turn lead to activity-dependent plasticity in S1 [37] via thalamocortical projections. Indeed, repeated noxious stimulation in healthy individuals can increase S1 GM volume [38]. With pain chronicity, thicker S1 cortex, particularly of the contralateral putative face region, may reflect enhanced intensity and localization of nociceptive information from the face. Increased S1 GM has previously been reported in chronic pain disorders involving the trigeminal system including migraine, temporomandibular disorder (TMD), and trigeminal neuropathic pain [12], [39], [40].

We further observed that TN patients had thinner cortex in the pgACC, aINS, and OFC compared to controls, a finding that has been reported in numerous chronic pain conditions [13]. These regions have been implicated in aspects of the cognitive-affective dimension of pain as well as other functions including attention, salience, interoception, and top-down mechanisms such as placebo analgesia, and pain modulation [17]. The pgACC is thought to contribute to pain unpleasantness, salience, and the regulation of emotional information [13], while the aINS has been implicated in salience as well as pain intensity and anticipation, and negative emotions such as anxiety [41]. The role of the OFC includes sensory, pain, and emotion regulation [42]. Less GM in these regions may then reflect the high degree of pain unpleasantness and/or emotional responses to TN or chronic pain in general, as similar findings have been reported in several chronic pain populations [13].

The mechanism of pain-related cortical thinning is not yet established. One possibility, as described in other patient populations, is that there are focal losses of cortical inhibitory interneurons, making these regions hyperactive [43]. In the chronic pain literature, evidence to support alterations in cortical inhibition comes from studies using transcranial magnetic stimulation (TMS) on patients with chronic pain [44]. By applying magnetic pulses to the cortex of chronic pain patients and measuring peripheral muscle activity, TMS studies have demonstrated changes in cortical inhibition mediated by gamma aminobutyric acid (GABA) receptors [44]. Although these studies have focused on the motor cortex, it is possible that similar mechanisms are taking place in other cortical regions and will be an avenue of future research [44]. An alternate explanation is that thinner cortex reflects pruning due to use-dependent synapse elimination, as associated with the development of cognitive abilities and behavior [45]. Thus, thinner cortex in these regions could indicate an enhanced or more efficient system for processing the affective dimension of pain.

Interestingly, the FP was thicker in TN patients; a finding also reported in TMD [12]. The precise function of the FP is not well understood, but it has been implicated in a number of complex cognitive functions including multitasking, monitoring, and evaluating expected outcomes [46]. It is possible that the thicker cortex in the FP may occur because there is a greater cognitive load associated with having TN. Future behavioral studies are thus needed to determine the role of cognitive factors on the structural abnormalities reported in the current study.

Similar to findings in migraine [47], another paroxysmal pain disorder, we report greater PAG volume in TN patients. It has been proposed that the descending pain modulatory system is altered in chronic pain such that there is either dysfunction in nociceptive inhibition, or enhancement in nociceptive facilitation [17]. Previous animal research has shown that PAG stimulation inhibits trigeminal nociceptive signals, suggesting that PAG abnormalities may lead to the disinhibition of trigeminal afferents [48]. Greater PAG volume may reflect activity-dependent increases in modulation because the system is ineffective at inhibiting nociceptive signals, or because there is a need to counter-balance the barrage of nociceptive input from the trigeminal nerve. Additionally, pain modulation can be influenced by several psychological factors such as attention, emotion, placebo, and anticipation via connections to cortical regions including the ACC, insula, and PFC and subcortical regions such as the amygdala [17]. This combined with a larger PAG volume may reflect differences in the psychological modulation of TN pain. In our study, we show these regions to be abnormal in TN patients. Additionally, and consistent with our results, there is right-lateralized noxious-evoked amygdala activity, possibly related to the emotional response to pain and pain modulation [49]. Although evidence for pain-related lateralization is controversial, right hemispheric lateralization using functional neuroimaging techniques has also been documented in several brain areas, regardless of the side of stimulation [50].

Consistent with our hypothesis, we report increased GM in motor regions. One characteristic of TN is that normally non-painful movements can elicit attacks of pain, so some patients attempt to restrict facial movements. This restriction has been observed in other facial pains such as TMD [15]. The Pain Adaptation Model [14], [15] proposes a framework for nocifensive behaviors. Specifically, pain leads to alterations in muscular activity aimed at limiting movements of an affected muscle by redistributing function and load. In the short term, this protects the system from further injury to support healing, but prolonged muscular alteration can lead to more pain and further peripheral damage. Greater GM volumes in these structures may represent compensatory/nocifensive strategies employed by TN patients to prevent and/or decrease their pain. Since facial movements such as talking and chewing are characteristically bilateral, the bilateral putamen and motor thalamus findings may represent bilateral compensatory motor behaviours. Additionally, larger basal ganglia volumes have been reported bilaterally in patients with migraine, an interesting finding given that migraine pain, like TN, can be paroxysmal involving the trigeminal nerve [51]. Importantly, M1 findings may also reflect alterations to the motor root of the trigeminal nerve and/or abnormal pain modulation, given the use of motor cortex stimulation as a treatment for neuropathic pain [52].

Some basal ganglia structures that were larger in TN patients have also been implicated in non-motor functions. For example, evidence suggests a role for the putamen in the sensory aspects of pain [53]. TN patients also had larger NAc volumes, which may reflect greater efforts to evaluate ongoing pain and/or predict future outcomes regarding pain [54].

Some previous studies of chronic pain have found correlations between pain duration and brain GM [10], [12], suggesting that long term pain drives neuroplasticity [38]. We did not find evidence for this relationship in our TN patients. However, TN is quite different since it consists of repetitive paroxysmal pain rather than the sustained ongoing pain characteristic of other neuropathic pains. Additionally, TN patients are heterogeneous with regard to the number of pain attacks they have per day, and thus the use of pain duration may not be an adequate index of total pain over time for the purposes of assessing pain-driven brain changes in this patient group. Future longitudinal studies are needed to explore this factor.

Additionally, nearly all of our patients were taking medication for TN, with the most common being the anticonvulsant, carbamazepine. Although the precise impact of these medications on brain morphology is not known, it is possible that they influence structural abnormalities. Future studies are needed to examine the effects of these drugs on brain GM.

Taken together, our study demonstrates for the first time, that patients with idiopathic TN have prominent GM abnormalities in brain regions involved in sensory-discriminative and cognitive-affective dimensions of pain, pain modulation, and motor function. Unlike other facial pains, TN patients have paroxysmal pain, frequently triggered by innocuous stimuli, and are without major sensory loss. The GM abnormalities reported in the current study likely reflect this unique symptomology. Understanding the contribution of central GM abnormalities in this population is a crucial step towards elucidating the mechanisms underlying this unique neuropathic pain.
